# Rapid Gamete Maturation and Social Modulation Shape Reproductive Dynamics in a Brood Parasitic Catfish

**DOI:** 10.1002/ece3.73017

**Published:** 2026-01-30

**Authors:** Holger Zimmermann, Radim Blažek, Matej Polačik, Anna Bryjová, Lukas Koch, Martin Reichard

**Affiliations:** ^1^ Institute of Biology University of Graz Graz Austria; ^2^ Institute of Vertebrate Biology Czech Academy of Sciences Brno Czech Republic; ^3^ Department of Botany and Zoology, Faculty of Science Masaryk University Brno Czech Republic; ^4^ Department of Ecology and Vertebrate Zoology University of Łódź Łódź Poland

**Keywords:** adaptation, brood parasitism, cuckoo catfish, reproductive competition, resource monopolization

## Abstract

Obligatory brood parasitism requires specific reproductive adaptations in brood parasites to successfully exploit host reproduction. A key challenge is to precisely synchronize parasite egg laying with egg laying of the host. The Lake Tanganyika cuckoo catfish ( *Synodontis multipunctatus* ) exploits the mouthbrooding behavior of cichlids by rapidly intruding during host spawning and adding its own eggs to host clutches. Because host reproduction is highly unpredictable, cuckoo catfish must respond quickly when host spawning occurs. Such a reproductive strategy suggests the presence of adaptations in their reproductive biology that allow them to be able to participate in upcoming host spawning opportunities. Using a series of linked laboratory experiments, we investigated the reproductive physiology and social modulation of gamete maturation in cuckoo catfish, as well as reproductive skew during parasitic spawning events. We found that males consistently produced sperm with little inter‐individual variation, indicating almost continuous readiness to reproduce. In contrast, females exhibited substantial variation in ovulation frequency and ovulated, on average, once a week. Social interactions significantly increased gamete maturation rates in both sexes, highlighting the role of group dynamics in facilitating reproductive readiness. Despite high gamete maturation rates in socially housed females, parasitism success during host spawning events was not predicted by prior gamete production frequency. Smaller and slimmer males achieved higher reproductive success, suggesting a possibility that agility and reduced anticipation of potential threat by the hosts may confer advantages under competitive conditions. Reproductive skew was low across the experiment, and most individuals participated in reproduction over the duration of the experiment. However, single spawning events were typically monopolized by a single pair. These findings demonstrate that cuckoo catfish reproduction is shaped by a combination of frequent gamete maturation, social facilitation of their reproductive physiology, and scramble competition over mating opportunities.

## Graphical abstract

Obligatory brood parasitism in the Lake Tanganyika cuckoo catfish requires rapid reproductive readiness to exploit unpredictable host spawning. Our experiments showed that males are almost continuously ready to reproduce, while females ovulate about once a week, with social interactions enhancing gamete maturation in both sexes. Parasitism success was driven by scramble competition and smaller males achieved higher success.

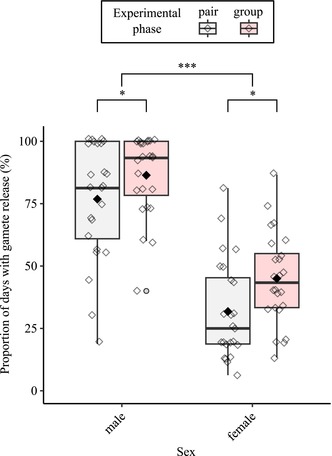

## 1 Introduction

Obligate brood parasites pass the workload of costly brood care entirely onto their hosts. Negative consequences for host fitness incite their responses to parasitism which often escalate in co‐evolutionary arms races of adaptations and corresponding counteradaptations of hosts and the brood parasites. These arms races represent textbook examples of co‐evolution and imply multiple layers of adaptations in the brood parasites' reproductive patterns (Davies 2000). The strongest co‐evolution between brood parasites and their hosts occurs at the front‐line of defense (Feeney et al. 2012), until the parasitic eggs are laid. One of the most critical steps towards successful brood parasitism is the synchronization of brood parasite reproduction (gamete maturation and oviposition) with the reproductive cycle of their hosts.

The Lake Tanganyika cuckoo catfish ( *Synodontis multipunctatus* ) is the only known non‐avian obligate brood parasite among vertebrates. Cuckoo catfish use mouthbrooding cichlids as foster parents to develop their eggs (Sato 1986; Reichard 2019) and face unique problems to locate suitable opportunities for parasitism and to synchronize their ovulation accordingly. Unlike in birds, where social monogamy prevails (Kempenaers 2022) and offspring are raised in refined and long‐lasting nests (Mainwaring et al. 2014), the typical hosts of cuckoo catfish do not form stable pairs. In avian brood parasites, parasitic females often monitor host nests for extended periods to properly time their egg deposition (Feeney et al. 2012). In many mouthbrooding cichlid species, males display in their short‐term breeding territories, and females visit one or several males to spawn (Sefc 2011). Male breeding territories are typically associated with a central cleaned patch of substrate where the males lead receptive females to spawn. The females are choosy and often inspect several males before mating, sometimes splitting their clutch among some of them. The spawning may hence last from a few minutes up to 1–3 h. In their natural environment, at least some host cichlids reproduce year‐round, with no specific breeding season [e.g., *Pseudosimochromis babaulti* (Kotrschal and Taborsky 2010), *Shuja horei* (Sefc 2011)]. Consequently, opportunities for cuckoo catfish parasitism are spatially and temporally unpredictable. These characteristics create conditions under which spawning resources (i.e., host spawning) appear undefendable for individuals or groups of cuckoo catfish and facilitate scramble competition over parasitism opportunities (Brown 1964; Parker 2000). Cuckoo catfish parasitism involves a mixed‐sex group of cuckoo catfish intruding on the cichlid spawning site (Blažek et al. 2021; Zimmermann et al. 2022; Zimmermann et al. 2025). One potential way to maximize reproductive success for individual catfish may be to establish dominance within their groups and suppress the reproduction among subordinate same‐sex group members (Beehner and Lu 2013; Freeman 2021). Previous studies on other fish species have shown that social interactions can play a crucial role in reproduction by altering the hormonal status of members of hierarchical groups (Maruska 2014) and, indeed, some form of reproductive suppression has been recorded from a captive cuckoo catfish group (Wisenden 1999).

Cuckoo catfish intrude on host spawning events in groups, overwhelm host defenses, and prey on some cichlid eggs (Blažek et al. 2021; Reichard et al. 2023). Simultaneously, some catfish release their own gametes. To protect her eggs, the host female hastily collects them and may include some parasite eggs in her clutch. To successfully parasitize their hosts, it is important for the cuckoo catfish to maximize the temporal availability of their gametes ready for spawning and therefore to keep inter‐spawning intervals short. An anecdotal observation of three cuckoo catfish (one male and two females) in an aquarium over a period of 18 months (65 parasitised clutches out of 133 host spawning events) suggested that the catfish are able to parasitize their hosts at a high frequency (several times a week), and sometimes even to parasitize the spawning of different hosts within the same day (Wisenden 1999). This is in contrast to other Lake Tanganyika *Synodontis* species. Aquarium populations of *S. petricola* and *S. polli* , two species that occur sympatrically with the cuckoo catfish in the wild, require an interval of several weeks between consecutive spawning events (pers. observation), despite histological examinations revealing no clear differences among the three species concerning their gamete maturation patterns (Dyková et al. 2024).

In this study, we investigated the cuckoo catfish potential to respond to parasitism opportunities by quantifying cuckoo catfish gamete maturation frequency. We hypothesized that (i) unpredictable parasitism opportunities favor the maintenance of a reproductive steady‐state in both cuckoo catfish sexes, (ii) the benefits of group intrusions during parasitism entail socially induced increases in gamete maturation frequency, and (iii) social interactions within groups can result in hierarchical structures with some individuals becoming dominant and experiencing increased gamete maturation, whereas other group members cease gamete production while becoming subordinate because of social suppression. We predicted that both male and female catfish are able to release gametes very frequently (several times a week) and that social interactions increase overall gamete maturation. Further, we used a controlled parasitism experiment and subsequent parentage analysis to investigate how gamete maturation frequency translates into cuckoo catfish reproductive success. We predicted that social interactions and the resulting hierarchical structure among group members affect gamete maturation and reproductive output.

## 2 Methods

Experimental cuckoo catfish were 7 years old (born in 2016) and were a mix of F1 and F2 generations of commercially imported wild‐caught parents (10 pairs, one population). All catfish used in the experiments were measured to the nearest mm prior to the first experimental phase (see below) but were only measured to the nearest mm and weighed to the nearest 0.1 g 4 months after the end of the second experimental phase (see below). No substantial change in size and weight of healthy fish was expected within a few months in catfish of that age (7 years). The experiments were conducted in two temporally separate phases. Throughout the entire experiment, water temperature was maintained at 27°C (±1°C), the dark—light regime was set to 11 h:13 h for all experiments, and catfish were fed ad libitum with a mixture of blood worms and commercial dry food pellets.

The first phase (Experiment 1) comprised monitoring of the individual frequency of gamete release in 24 pairs of cuckoo catfish under two different conditions. First, all fish were housed in pairs (1‐pair treatment, no intrasexual competition, 16 monitoring days from 16.11.2020 to 8.1.2021 in 24 tanks with a volume of 54 L) (Experiment 1A). Second, the same set of fish was housed in a treatment as a group of three pairs (3‐pairs treatment, with intrasexual competition, 15 monitoring days from 12.2.2021 to 2.4.2021, 8 tanks with a volume of 108 L) (Experiment 1B). The tanks were equipped with aquarium gravel (grain size 4 mm), internal filtration, and two (1‐pair treatment) or four (3‐pairs treatment) clay pot halves as shelters. Across both phases, all males and females were captured twice a week and monitored for gamete release by gently pressing the belly towards the anal fin. Given that fish were tested twice a week rather than daily (to reduce handling stress), eggs obtained by striping were recorded irrespective of whether they appeared viable (freshly ovulated, spherical, and light yellow) or non‐viable (either unripe or overripe, deformed, and whitish), because the presence of unripe or overripe eggs indicates ovulation temporally close to the day of striping. Sperm was clearly visible on the genital papilla and classified as either present or absent.

To verify the results of egg maturation frequency obtained through manual gamete striping, we set up a separate experiment to test the temporal dynamics of natural egg release (Experiment 2). We used 10 cuckoo catfish pairs not used in the main experiment and kept them in pairs in separate 54 L tanks. The tanks had a fine false mesh bottom (mesh 4 by 4 mm) and were equipped with internal filtration, a clay pot half as a shelter, and a glass petri dish (94 × 16 mm) as a feeding spot positioned on the false mesh bottom to ensure optimal food presentation and prevent food decomposition in the section below the mesh. Fish were initially kept without any additional stimulus for 34 days (09.02.2021–14.03.2021) and then with the addition of host cues ( *Astatotilapia burtoni* male placed in the tank) for another 33 days (15.03.2021–17.04.2021). As female cuckoo catfish regularly release their over‐ripe eggs when spawning with hosts is not possible (personal observation by authors), we monitored female egg production by daily counting the eggs collected under the false bottom as a sign of female ovulation. All eggs were collected and checked for maturity level (2321 eggs from 204 different occasions; all were over‐ripe, whitish and of soft consistency).

The second phase (Experiment 3) (30.9.2022–6.6.2023) tested how these individual gamete release rates translated into reproductive success in the presence of hosts. The experiments were conducted in large aquaria (405 L; length 150 cm, depth 60 cm, height 45 cm) equipped with internal filtration, standard aquarium gravel, and two large shelters made of clay pots. Host cichlids were laboratory populations of either *Jabarichromis* (*Gnathochromis*) *pfefferi* (10 males and 15 females per experimental tank, JP) or *Maylandia zebra* ‘red’ (6 males and 15 females per experimental tank, MZ) housed in mixed‐sex groups in 405 L separate housing tanks prior to the experiment. We retained the same cuckoo catfish group compositions as in the 3‐pairs treatment of the first phase (i.e., Experiment 1B, 3 male and 3 female cuckoo catfish per experimental tank). We inspected all experimental tanks for any mouthbrooding host females three times a week. We captured any mouthbrooding host using a hand net and gently removed her clutch by using a jet of water from a Pasteur pipette. Every clutch was inspected for the presence of cuckoo catfish eggs (small (2.7 mm) and spherical in contrast to larger and oval host eggs). All cuckoo catfish eggs were then incubated in egg tumblers (Zimmerman et al. 2023) for 6 days before they were euthanized in a bath of ice and preserved in 99% ethanol for further genetic parentage analysis. Three of the eight catfish groups did not produce a reasonable number of clutches (*n* < 3) and were excluded from the parentage analysis. In another experimental tank, one male catfish died during the experiment, and no final standard length measurement was taken, prompting us to exclude all males of this group from analyses where standard length (mean‐centred within tanks) was used as a predictor of male reproductive success. One additional tank with a group of catfish not used in Experiment 1 nor Experiment 2 (but otherwise kept under identical conditions as described for Experiment 3) provided a reasonable number of clutches. We estimated parentage in those clutches and included the resulting parentage data in the appropriate analyses (note that no information on gamete release frequency was available for this group).

### 2.1 Ethics

Research adhered to all national and institutional animal care and use guidelines (permit No. CZ62760203) and was approved by Ethical Boards of the Institute of Vertebrate Biology and the Czech Academy of Sciences (approval No. 32‐2019).

### 2.2 Genetic Parentage Analysis

We obtained 244 cuckoo catfish embryos from 41 clutches from a total of 6 experimental groups (15, 7, 6, 7, 3, and 3 clutches, respectively). We used the GeneJET Genomic DNA Purification Kit (Thermo Scientific) following the manufacturer's protocol to obtain DNA from 6 days old cuckoo catfish embryos. Additionally, we obtained DNA from fin clips of the cuckoo catfish lab population (*n* = 167 individuals) to estimate population allele frequencies. For microsatellite analysis, we used a total number of 17 primer pairs organized into three sets (multiplexes) according to their expected amplicon size (Zimmermann et al. 2025). The reverse primers were M13 (GGA AAC AGC TAT GAC CAT), CAG (CAG TCG GGC GTC ATC) or T3 (AAT TAA CCC TCA CTA AAG GG) tailed and labeled with fluorescent dye (VIC, FAM, and NED) during PCR according to a previously used multiplex organization (Zimmermann et al. 2025). PCR was performed in 10 μL volume, containing 1 μL of DNA, 5 μL of Multiplex PCR Master Mix (Qiagen), and Primer Mix 1× (0.1–0.75 μM forward primers and 0.1–1.1 μM labeled tails, and 0.01–0.075 μM reverse primers). The PCR protocol consisted of an initial denaturation step at 95°C for 15 min, followed by 30 PCR cycles (95°C for 30 s, 57°C for 90 s, 72°C for 30 s) and 8 PCR cycles with reduced annealing temperature (95°C for 30 s, 53°C for 90 s, 72°C for 30 s), and a final extension at 68°C for 10 min. Fragment sizes were scored against the internal size standard (GeneScan LIZ‐500) using an ABI 3130 Genetic Analyser (Applied Biosystems, Foster City, California, USA).

### 2.3 Assignment of Parentage

Catfish offspring and the reference population sample were genotyped using the GeneMapper software (version 3.7, Applied Biosystems). We used the software ‘identity4’ (Wagner and Sefc 1999) to estimate population allele frequencies from the 167 samples of adult cuckoo catfish. We used the R packages ‘adegenet’ and ‘pegas’ (Jombart 2008; Paradis 2010) for a quality check of the marker set (Table S1, Figure S1). Because our test population consisted of a relatively small number of putatively related catfish, deviations from Hardy–Weinberg equilibrium were to be expected. However, our study design (i.e., a set of candidate parents with known genotypes) provided us with full control over genotype combination within the experimental groups, and the application of a large set of 17 microsatellite loci allowed for unambiguous assignment of individual offspring to their parents. Parentage analysis was performed in COLONY 2.0 (Jones and Wang 2010) for each experimental group separately, and included the genotypes of six experimental catfish adults from a particular group as a set of candidate parents. The COLONY output was split to individual clutches collected from host females, resulting in a set of full‐ and half‐sib clusters for each spawning. The COLONY results for each clutch were carefully checked manually, and deviations from the known set of parental genotypes were re‐genotyped to eliminate any genotyping errors.

### 2.4 Statistical Analysis

We used R v. 4.5.1 (R Core Team 2025) for all statistical analyses. Generalized Linear Mixed‐effects Models (GLMM) were fitted using the R package ‘glmmTMB’ (Brooks et al. 2017). Model fits were evaluated using the functions ‘testDispersion’ and ‘simulateResiduals’ [R package ‘DHARMa’ (Hartig 2021)].

### 2.5 Frequency of Gamete Release (Experiments 1 and 2)

To examine the effects of sex and treatment on the gamete production (Experiment 1), we fitted a GLMM with beta‐binomial error distribution (*n* = 24 males and 24 females) to account for over‐dispersed data. We included the percentage of days with gamete release (coded as ‘days with gamete release’ versus ‘days without gamete release’) as the response variable and sex (male or female), treatment (1‐pair or 3‐pairs), and catfish standard length (mm) as predictor variables. We considered that the treatments may have affected the sexes differently (interaction sex * treatment), and that standard length may have sex‐specific effects (interaction sex * standard length) but removed both from the final model as they had no significant effects. We also applied random intercepts for catfish ID and experimental tank ID to account for non‐independence of data within the same tanks and from the same cuckoo catfish but excluded tank ID from the random structure because it explained near‐zero variability (< 0.0001). Additionally, we compared catfish clutch sizes between 1‐pair (Experiment 1A) and 3‐pairs treatment (Experiment 1B) to see whether social interactions with same‐sex fish affected egg production. For this analysis, we excluded all cases with no eggs during striping for each female since we were specifically interested in the clutch size. Because the data violated assumptions for fitting a regression model (e.g., homogeneity of variances), we used a Wilcoxon signed‐rank test using the mean clutch size per catfish female paired for both experimental phases.

To test for effects on the difference in gamete release between the two treatments (especially the effect of relative standard size), we first calculated the log‐ratio of the proportions of gamete release from 3‐pair and 1‐pair phase [log (% 3‐pair/% 1‐pair)]. Then, we fitted a GLMM with Gaussian error distribution (*n* = 21 males and 21 females) including the log‐ratio proportion as response variable, and sex and standard length (standardized by mean‐centering within the pooled males and females of each group) as predictor variables. We fitted ‘group ID’ as a random intercept to the model to account for the non‐independence of data obtained within groups and a random intercept for catfish ID to account for the repeated sampling of the same fish to create the proportions of gamete release. We removed the random intercept for group ID from the final model because it explained near‐zero variance (< 0.0001). We also considered that gamete release frequency in males and females may be affected by their body size (i.e., standard length) differently but removed the non‐significant interaction (sex * standard length) from the final model.

To test whether the presence of host cues affects the frequency of egg release (Experiment 2), we applied a GLMM on the egg release data obtained during the false mesh bottom experiment. We fitted the model with a negative binomial error structure to account for data overdispersion. We used the number of days when the eggs were released for each female as a response variable and the experimental conditions (host cues or no host cues) as a binary predictor variable. We also included the female ID as a random intercept to account for the non‐independence of data obtained from the same female. Similarly, to examine the effects on the number of eggs released during this experiment, we fitted a GLMM with a negative binomial error distribution (nbinom1). We included individual daily egg counts as a response variable, and treatment (presence or absence of host cues) and female standard length (mm) as predictor variables. Again, we included female ID as a random intercept to account for the non‐independence of egg counts from the same females.

### 2.6 Parasitism Experiment (Experiment 3)

To investigate catfish reproductive skew across and within groups, we calculated the index of relative resource monopolization (*Q*, Ruzzante et al. 1996). The *Q* expresses the distribution of reproductive success (calculated for each sex separately) as the variance in cuckoo catfish parentage found within host clutches in relation to the potential maximum variance (calculated as: (VAR − MEAN)/((*n* * MEAN^2) − MEAN); where VAR is the variance in parental success, *n* is the number of potential parents, and MEAN is the potential mean parental success). *Q* = 0 denotes equally distributed reproductive success for each sex within each group, and *Q* = 1 denotes complete monopolization. First, we calculated *Q* for the entire set of catfish offspring in each experimental tank to estimate overall reproductive skew (*Q*_TOTAL_) in that replicate. Then, we calculated *Q* for each clutch separately (for clutch size > 1 egg, *Q*_CLUTCH_), because the individuals within groups could alternate in spawning dominance, and that information would be lost when data are analyzed only as the sum of reproductive success over the entire period. Hence, *Q*_TOTAL_ and *Q*_CLUTCH_ describe different levels of reproductive monopolization—at the long term and at the level of individual clutches.

Additionally, we fitted a GLMM applying an ordered beta regression (Kubinec 2023) to test whether covariates affect reproductive skew. We used the *Q* indices calculated for each spawning (*Q*_CLUTCH_) as the response variable (*n* for females = 34, *n* for males = 29), sex, within‐group‐standardized length differences (sex specific), and host species (MZ and JP with 59 and 4 data points for *Q*_CLUTCH_, respectively) as predictor variables. We also included catfish clutch size (for each clutch) as an additional predictor variable because we expected a higher likelihood of multiple parentage in larger catfish clutches. We also included the ID of each individual spawning nested within tank ID as a random intercept to account for the non‐independence of data.

To test for effects on the number of cuckoo catfish offspring parented by individual catfish, we fitted a GLMM with a beta‐binomial error distribution to account for data overdispersion (*n* = 186 individual data points from 31 clutches across 4 experimental tanks). We included the proportion of cuckoo catfish offspring each adult catfish parented in the particular spawning (coded as ‘offspring parented’ versus ‘offspring not parented’) as the response variable and the catfish sex, the Fulton's condition factor (*K*_*C*_, 100 * weight[gram]/total length[cm]^3), the mean‐centred total length (centred at the intrasexual mean within groups), and the percentage of days gametes were released during the 3‐pairs treatment of the ovulation frequency experiment (further ‘gamete production’) as predictor variables. Further, we fitted spawning ID nested within tank ID as a random intercept to correct for non‐independence of data within individual catfish clutches in each experimental tank. The initial model fit also accounted for potential intersexual variation in these effects by fitting the pairwise interactions of sex with the other three predictor variables. The interaction between sex and gamete production was not significant and therefore dropped from the final model. A significant effect of the interaction between sex and Fulton's condition factor and a trend for a significant effect in the interaction between sex and total length indicated different effects of these variables on the number of parented offspring for each sex. Therefore, we subsequently ran the same model fit as described above for each sex separately. As body condition may correlate with length, we checked the Variance Inflation Factors (VIFs) for the three model fits using the function ‘check_collinearity’ [R package ‘performance’ (Lüdecke et al. 2021), all < 2.4].

## 3 Results

### 3.1 Gamete Production Frequency Experiments (Experiments 1A, 1B, and 2)

**FIGURE 1:**
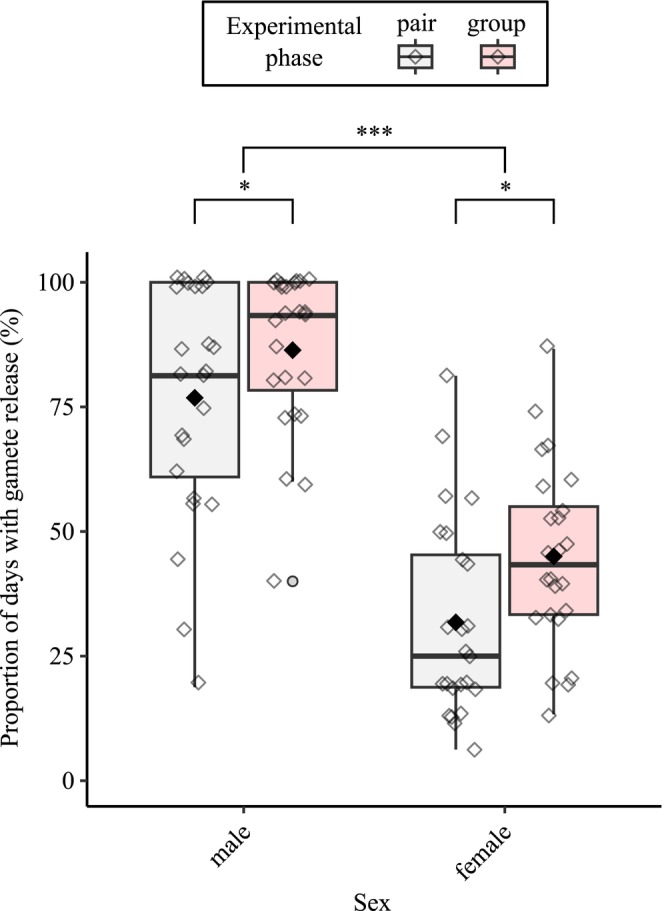
Proportion of days cuckoo catfish gave gametes in the first phase experiment (Experiment 1). Box plots show medians (thick vertical lines), first and third quartiles (boxes), and the range of data within 1.5 interquartile distances above and below the interquartile (whiskers). Points represent individual data points (open diamonds) and means (filled diamonds). *p < 0.05, ***p < 0.001.

When kept in pairs (Experiment 1A), individual cuckoo catfish gave gametes, on average, every second monitoring day (mean ± SD: 54.3% ± 31.4% of days). This percentage was significantly higher during the second phase (Experiment 1B) when the cuckoo catfish were kept in 3‐pairs groups (65.7% of days ± 27.0%; GLMM: est. = 0.58, SE = 0.21, *z* = 2.73, *p* = 0.006; Figure 1). Irrespectively of experimental phase (1‐pair and 3‐pairs), males gave gametes on significantly more monitoring days than females (males = 81.6% ± 20.6% of days, females = 38.4% ± 20.0% of days; GLMM: est. = 2.14, SE = 0.26, *z* = 8.25, *p* < 0.001; Figure 1). Body size (represented as standard length) had no significant effect on the gamete availability of the catfish (GLMM: est. = 0.002, SE = 0.02, *z* = 0.11, *p* = 0.92).

Gamete production increased from 1‐pair to 3‐pairs treatment (Experiments 1A and 1B) on average by 11.4% (i.e., individual catfish gave gametes on 11.4% more days during the 3‐pairs treatment than during a 1‐pair treatment). The increase in gamete production during the 3‐pairs treatment was significantly higher in females than in males (mean increase of days ± SD, males = 9.6% ± 27.4% of days, females = 13.2% ± 22.8% of days; GLMM: est. = −0.36, SE = 0.17, *z* = −2.16, *p* = 0.0309). Further, there was a non‐significant tendency for relatively smaller individuals (standardized within sex and replicate) to experience a smaller increase (or even a decline) in gamete production between Experiment 1A and Experiment 1B (GLMM: est. = 0.033, SE = 0.02, *z* = 1.88, *p* = 0.0598). Additionally, monitored clutch sizes remained stable between treatments with a median of 7 eggs in the 1‐pair treatment (interquartile range, IQR = 2 to 24 eggs) and a median of 10 eggs in the 3‐pairs treatment (IQR = 2 to 27 eggs; Wilcoxon signed rank test: *n* = 270 spawning events, *V* = 187.5, *p* = 0.2904).

**FIGURE 2:**
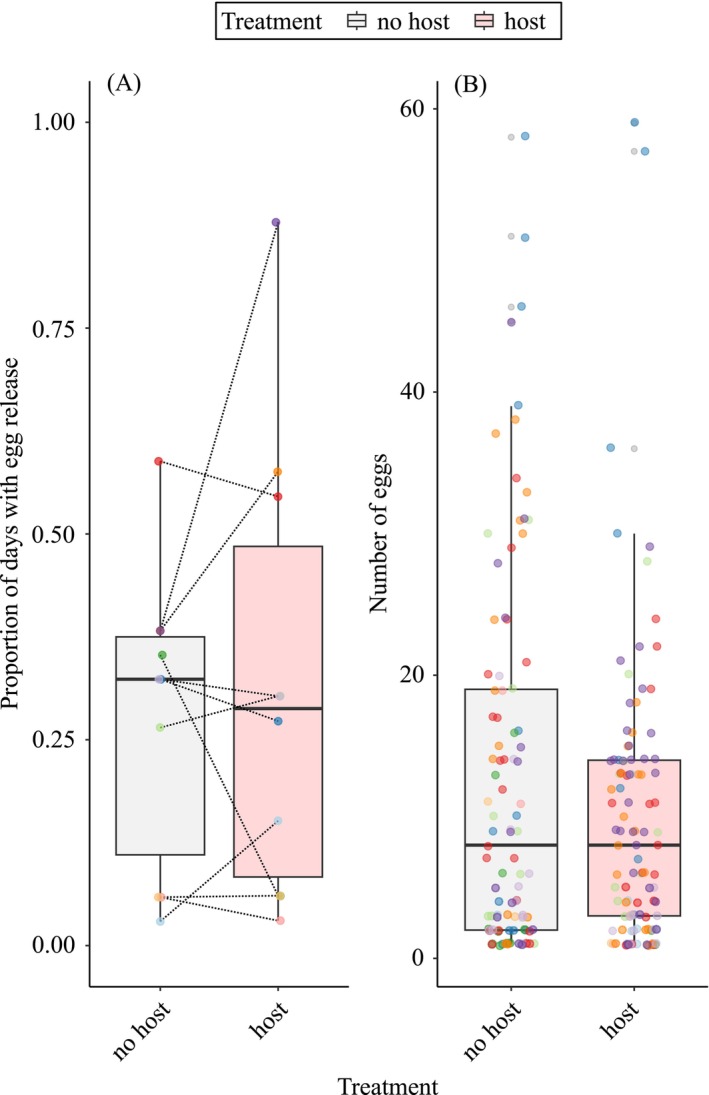
Proportion of days with egg release (A) and the number of eggs females released (B) with and without host cues. Egg release was monitored for 10 females for 34 days (no host treatment) and 33 days (host treatment). Dotted lines in (A) indicate paired data points. Colors indicate individual females.

Natural egg release (Experiment 2) over the mesh false bottom was observed on average at 27.6% of days when host cues were absent (mean ± SD, 9.4 ± 6.1 days over the period of 34 observation days) and at 31.8% of days with host cues present (10.5 ± 9.1 days over the period of 33 observation days, Figure 2A). This was slightly less often than when the eggs were manually stripped from females in 1‐pair and 3‐pairs experiments (31.8% and 45.0%, respectively) but can be considered comparable given the daily sampling regime in Experiment 2. There was no significant difference in the frequency of natural egg production between treatments (GLMM: est. = 0.07, SE = 0.21, *z* = 0.34, *p* = 0.737, Figure S2). The number of eggs released was highly variable among females (mean ± SD per treatment: without host cues = 10 ± 10 eggs, with host cues = 13 ± 13 eggs; Figure 2B) and neither female size (standard length in mm) nor the presence of host cue had significant effects on the number of released eggs (GLMM: female size, est. = 0.01, SE = 0.01, *z* = 0.42, *p* = 0.675; treatment, est. = 0.11, SE = 0.12, *z* = 0.91, *p* = 0.363; Figure S2).

### 3.2 Parasitism Experiment (Experiment 3)

**FIGURE 3:**
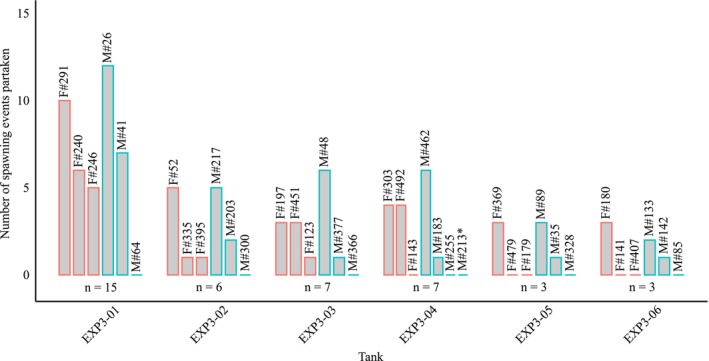
Individual participation in parasitic spawning events by cuckoo catfish. Individual IDs start either with F for females (red contour color) or M for males (blue contour). * indicates one deceased male that was replaced during the experiment.

**FIGURE 4:**
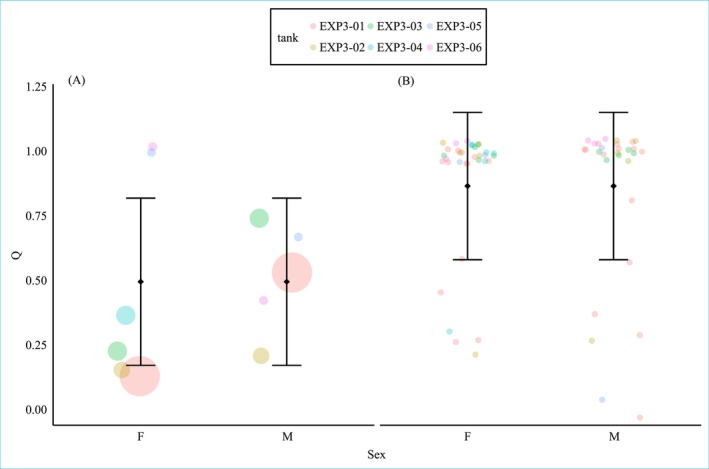
Indices of relative resource monopolization (Q) calculated (A) for the sum of reproduction within each experimental tank and (B) for each cuckoo catfish spawning separately. Black dots and vertical bars represent group means ± SD Different colors represent the experimental tanks. In (A), the size of the colored points represents the different numbers of spawning events.

Parentage analysis revealed that within‐group reproductive success varied among cuckoo catfish individuals but, overall, was not strongly monopolized by particular individuals. Two of three males participated in reproductive success in each experimental group (Figure 3). In females, the distribution of reproductive success varied across groups, and the number of successful females in a group was positively correlated with the number of spawning events in the groups (3 females in 3 groups, two females in 1 group, and a single female in 2 groups; Figure 3). The overall monopolization of spawning events, expressed by the index of relative resource monopolization *Q*_TOTAL_, was on average 0.51 for males (SD ±0.22) and 0.48 for females (±0.41, Figure 4A). 83% of clutches (34 out of 41, Table S2) were produced by a single pair of catfish. A detailed analysis calculated over separate clutches (*Q*_CLUTCH_) revealed that specific individuals (pairs) commonly monopolized reproduction of particular clutches (Figure 4B). This translated to higher *Q*_CLUTCH_ than *Q*_TOTAL_ (*Q*_CLUTCH_ in males = 0.84 ± 0.32, *Q*_CLUTCH_ in females = 0.89 ± 0.26) and the odds for monopolizing a spawning did not differ significantly between males and females (GLMM: est. = 0.14, SE = 0.18, *z* = 0.78, *p* = 0.434). Neither within‐group standard length variation nor the catfish clutch size had significant effects on the reproductive skew within groups (GLMM on *Q*_CLUTCH_: standard length, est. = −0.03, SE = 0.03, *z* = −1.1, *p* = 0.273; clutch size, est. = 0.03, SE = 0.05, *z* = −0.63, *p* = 0.526). There was no effect of host species on the odds for monopolization (GLMM on Q_CLUTCH_: est. = 0.54, SE = 1.42, *z* = 0.38, *p* = 0.705).

**FIGURE 5:**
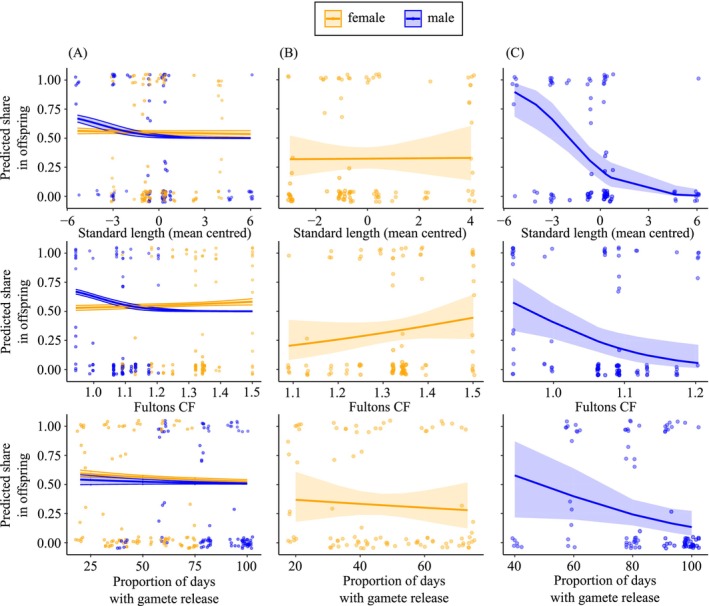
Effects of mean‐centred standard length, Fulton's condition factor, and gamete release during 3‐pairs experiment on the individual share of cuckoo catfish in reproduction as predicted by GLMMs fitted for both sexes (A) and for females (B) and males (C) separately. Blue = male, orange = female. *N* = 186 individual shares from 31 spawning events in 4 experimental tanks.

Average paternity shares were slightly, but significantly, higher than average maternity shares (GLMM: est. = 18.03, SE = 5.37, *z* = 3.36, *p* = 0.0008). Standard length, condition, and the frequency of gamete production during the 3‐pairs experiment were not significant predictors of individual reproductive success (GLMM: mean‐centred Standard length, est. = −0.06, SE = 0.12, *z* = −0.54, *p* = 0.5883; Fulton's CF, est. = 2.33, SE = 2.05, *z* = 1.14, *p* = 0.2543; gamete production, est. = −0.02, SE = 0.01, *z* = −1.70, *p* = 0.0883; Figure 5A). Significant interaction terms indicated negative effects of Fulton's CF and relative (mean‐centred) standard length on the paternity share in males (GLMM: sex_male_ * Fulton's CF, est. = −16.07, SE = 5.06, *z* = −3.18, *p* = 0.0015; sex_male_ * mean‐centred SL, est. = −0.58, SE = 0.21, *z* = −2.72, *p* = 0.0064).

A sex‐specific separate GLMM for females showed that the distribution of maternity within spawning events was not significantly affected by any of the fitted predictor variables (all *p*‐values > 0.177; Table S3; Figure 5B). In contrast, a male‐specific GLMM confirmed that the distribution of paternity during spawning events was biased towards smaller males, males with relatively lower body condition (i.e., slim males), and, additionally, towards males which did produce gametes with a lower frequency relative to other males in the experiment during the 3‐pairs phase of the gamete production experiment (GLMM for males: mean‐centred SL, est. = −0.63, SE = 0.16, *z* = −4.09, *p* < 0.0001; Fulton's CF, est. = −12.05, SE = 4.37, *z* = −2.76, *p* = 0.0058; gamete production, est. = −0.04, SE = 0.02, *z* = −2.04, *p* = 0.0414; Table S4; Figure 5C).

## 4 Discussion

Synchronizing reproduction with the reproduction of their host can be difficult for brood parasites, especially when reproductive resources are distributed unpredictably. In this study, we examined the potential of the brood parasitic cuckoo catfish to rapidly respond to the unpredictable occurrence of host spawning events through a series of linked laboratory‐based experiments to monitor the frequency of egg and sperm release and reproductive skew. Our results indicate that both male and female catfish are capable of producing mature gametes at high frequency. There was only a negligible variation in sperm availability among individual males, and they appeared to be almost always ready for gamete release. In contrast, females showed a high inter‐individual variation in ovulation rates. Social interactions within groups generally increased the availability of gametes in both sexes. Reproduction within catfish groups was not monopolized by particular individuals over time, but the potential for monopolization of specific spawning events was high in both sexes.

The results of the present study suggest clear adaptations of the reproductive physiology of cuckoo catfish to brood parasitism. Males apparently provided sperm over the entire monitoring period, possibly enabled by the generally good storage capabilities of mature sperm (Cattelan and Gasparini 2021). Females typically produced eggs every second or third monitoring day, indicating approximately one ovulation per week (on the basis of the short time window in which viable eggs are present in females) (Dyková et al. 2024). Batch spawning (also termed fractional spawning) represents a common reproductive strategy in fish (Wootton and Smith 2015) but is uncommon in *Synodontis* catfishes. Strong seasonal reproductive patterns, with synchronous release of large egg clutches, are known from several riverine *Synodontis* species (Halim and Guma'a 1989; Lalèyè et al. 2006; El‐Kasheif et al. 2012; Skelton 2024) and there is evidence that one Lake Tanganyika *Synodontis* species also exhibits seasonal reproduction (*S. irsacae*, Dyková et al. 2024). A histological examination of gonads suggests that the cuckoo catfish and the other two sympatric congeners ( *S. petricola* , *S. polli* ) reproduce year‐round (Dyková et al. 2024). Yet, cuckoo catfish adaptation to brood parasitism is characterized by a unique production of an order of magnitude fewer eggs at a time compared to the two other species (unpublished data). Further, *S. petricola* and *S. polli* females require several weeks to recover to spawning condition (pers. obs. on the basis of lab populations). Hence, the ability of the cuckoo catfish to spawn at short intervals seems unique among *Synodontis* and indicates its adaptations in reproductive physiology that enable cuckoo catfish to rapidly respond to forthcoming host spawning events.

Interactions with other cuckoo catfish females increased the frequency of egg production. In our experiment, females housed in groups of three pairs increased their gamete production compared to pair housing conditions. This further underpins the importance of social groups for cuckoo catfish reproduction. The cuckoo catfish commonly intrude on host spawning events in groups (Blažek et al. 2021; Zimmermann et al. 2022; Zimmermann et al. 2025), but our results represent the first evidence to link gamete maturation rate to social interactions in this species. Social interactions have a crucial influence on the endocrine and neuroendocrine status of fishes (Maruska 2014; Galhardo and Oliveira 2014). Most prominently, these effects have been demonstrated in the relationship between reproductive status and dominance status of males (Maruska 2014; Galhardo and Oliveira 2014; Maruska et al. 2022; Benvenuto and Lorenzi 2023) but may also have a crucial impact on female egg maturation. In the cooperatively breeding cichlid *Neolamprologus pulcher* , females adjust egg size to group size by producing smaller eggs when they live in larger groups (Taborsky et al. 2007). Apple maggot flies ( *Rhagoletis pomonella* ) and German cockroaches ( *Blattella germanica* ) increase their oviposition activities when exposed to social interactions with other females (Prokopy and Reynolds 1998; Uzsák and Schal 2013). In *Drosophila melanogaster* , females advance their ovulation in the presence of other females to minimize competition between their offspring and those of conspecifics (Bailly et al. 2023). Hence, an increase in the frequency of egg production as a response to the presence of competing females appears a convincing scenario for the cuckoo catfish. This adds to previously suggested benefits of group spawning in the cuckoo catfish, such as easier overcoming of host defenses during spawning and greater confusion in the host pair, facilitating the host acceptance of the parasite eggs (Blažek et al. 2021; Zimmermann et al. 2025). Consequently, groups may not only increase competition among females but also increase perceived parasitism perspectives, which may result in more frequent egg maturation. Cuckoo catfish spawning situations are likely competitive (Blažek et al. 2021). All cuckoo catfish compete to consume energetically rich host eggs. Males compete for fertilization success, and females compete for limited space in the host females´ buccal cavity (Blažek et al. 2021; Zimmermann et al. 2025). Hence, it would be beneficial for female cuckoo catfish to also increase the number of eggs produced because they have only very limited control over which eggs will be adopted by the host female. Additionally, an increase in the number of eggs could increase the chance of egg adoption before the limited space in the host buccal cavity is filled. However, females did not increase clutch sizes as a response to intrasexual interactions in the present study.

One surprising outcome of the present study is that a high rate of gamete maturation during the 3‐pairs phase (Experiment 1B) of the study has not translated to higher reproductive success during the parasitism experiment (Experiment 3). There was no link between egg maturation frequency in the 3‐pairs phase and parasitism success of individual females. In males, a relatively lower frequency in sperm availability during Experiment 1 resulted in the highest share of offspring when the cuckoo catfish reproduced with hosts. Such successful males were typically smaller and, as indicated by their lower Fulton's CF, slimmer compared to their within‐group male competitors. Several factors may explain this unexpected outcome. First, with few exceptions, even males with a relatively low frequency in visible sperm release provided sperm on most sampling days (Figure 1), and variation in sperm availability in our experiment may have been limited as a predictor for parental share success. Second, smaller males might have avoided host defenses more successfully. Host parents may anticipate larger males as a higher threat and, consequently, focus their defense more on intruders in better condition, leaving smaller males with higher chances for successful mating during group intrusions. Moreover, smaller and slimmer males may be able to utilize the restricted space created by the intrusion of several cuckoo catfish more efficiently than larger, more robust males. For example, in some poecilid fishes, smaller males may outcompete larger males through sneaky copulations despite larger males having an advantage in male–male competition as well as in female choice for large males (Bisazza and Pilastro 1997). Additionally, since we did not assess sperm motility, the number of cells, or other ejaculate traits, some observed variation in paternal success may be attributed to differences in sperm performance.

Reproductive skew was limited, with more individuals successfully participating in reproduction than we predicted. We hypothesized a dominance hierarchy and consequent share in reproductive success (Wisenden 1999). In species living in reproductive groups, reproductive skew is common, especially when the environmental or demographic conditions for successful reproduction favor group aggregation (Nonacs and Hager 2011). It may ultimately lead to reproductive suppression in same‐sex individuals when within‐group competition is high (Clutton‐Brock and Huchard 2013). This pertains to cuckoo catfish reproduction as host spawning events are unpredictable resources, and the catfish must overcome host defenses before they can spawn (Zimmermann et al. 2025; Johnson et al. 2002). Our experimental results discount a major effect of the host spawning monopolization in the long term, but indicate a high level of pair monopolization of a particular host spawning. A previous anecdotal observation of putative reproductive suppression between two females (Wisenden 1999) is not supported in the present study as a common phenomenon. Although most catfish individuals participated in reproduction over the long experimental period, most individual clutches were parented by a single pair of catfish. Hence, the potential for monopolization was high for separate spawning bouts. Overall, this indicates very dynamic interactions within the group during spawning activities. Although many group members succeed in reproduction overall, single spawning events were dominated by single pairs. This conforms to the previous reports of varying participation in intrusions to host spawning events (Zimmermann et al. 2022). The cuckoo catfish intrusions involve host egg predation and parasitic egg deposition, thereby providing trophic and reproductive benefits for the intruding groups. Consequently, individual motives for intrusions can vary, and it seems feasible that some individuals join intrusions to feed rather than to reproduce. In the absence of strict dominance hierarchies (including the reproductive suppression of subordinate group members), motives for individual participation in intrusions may strongly depend on the availability of mature gametes.

Collectively, our findings suggest that cuckoo catfish exhibit high frequencies of gamete maturation and are well adapted to their brood parasitic mode of reproduction, including temporally unpredictable spawning prospects. Marked differences in gamete maturation frequency compared to both riverine and sympatrically occurring Lake Tanganyika *Synodontis* species highlight the importance of ovulation synchrony with emerging reproductive opportunities in brood parasites. Interestingly, although individual parasitism events were often monopolized by single cuckoo catfish pairs, the potential for monopolization was weak over the long term. This pattern may result from scramble competition dynamics, where varying participation of cuckoo catfish in intruding groups during each host spawning event likely depends on individual gamete maturation frequency and reduces long‐term consistency in dominance.

## Author Contributions

**Holger Zimmermann:** conceptualization (supporting), data curation (equal), formal analysis (lead), funding acquisition (supporting), investigation (equal), methodology (supporting), project administration (equal), validation (equal), visualization (lead), writing – original draft (equal), writing – review and editing (equal). **Radim Blažek:** conceptualization (equal), data curation (equal), investigation (equal), methodology (equal), writing – original draft (supporting), writing – review and editing (supporting). **Matej Polačik:** conceptualization (equal), data curation (equal), investigation (equal), methodology (equal), writing – original draft (supporting), writing – review and editing (supporting). **Anna Bryjová:** investigation (supporting), methodology (supporting), writing – review and editing (supporting). **Lukas Koch:** investigation (supporting), methodology (supporting), writing – review and editing (supporting). **Martin Reichard:** conceptualization (equal), funding acquisition (lead), project administration (equal), supervision (lead), writing – original draft (equal), writing – review and editing (equal).

## Funding

This work was supported by the Austrian Science Fund (FWF, grants https://doi.org/10.55776/J4584 and https://doi.org/10.55776/PAT1680024), Grantová Agentura České Republiky (21‐00788X).

## Conflicts of Interest

The authors declare no conflicts of interest.

## Supporting information


**Data S1:** ece373017‐sup‐0001‐Supinfo.pdf.

## Data Availability

Analyses can be reproduced using the data and code provided at Figshare: https://doi.org/10.6084/m9.figshare.29900900.
