# Supplementary material for: Rapid Gamete Maturation and Social Modulation Shape Reproductive Dynamics in a Brood Parasitic Catfish
Source: Ecol Evol. 2026 Jan 30;16(2):e73017. doi: 10.1002/ece3.73017 (PMC12856514; doi:10.1002/ece3.73017)
Supplement: Supplementary file 1 — Data S1: ece373017‐sup‐0001‐Supinfo.pdf. [file ECE3-16-e73017-s001.pdf]

Supplementary figures and tables to the publication ‘Rapid gamete maturation and social modulation shape reproductive dynamics in a brood parasitic catfish’.

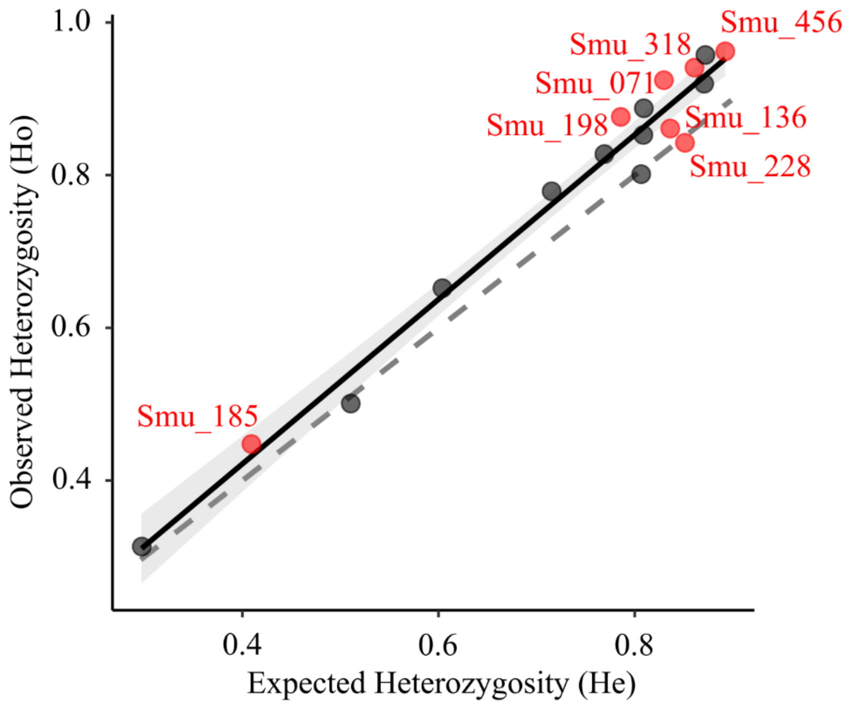

Supplementary Figure S1: Comparison between expected (He) and observed (Ho) heterozygosity of 17 microsatellite loci used for a parentage analysis. Red coloured loci deviated significantly from Hardy-Weinberg equilibrium. Grey dashed line represents equal He and Ho. Solid black line represents actual relationship between He and Ho as predicted by a linear regression (± 95% confidence interval).

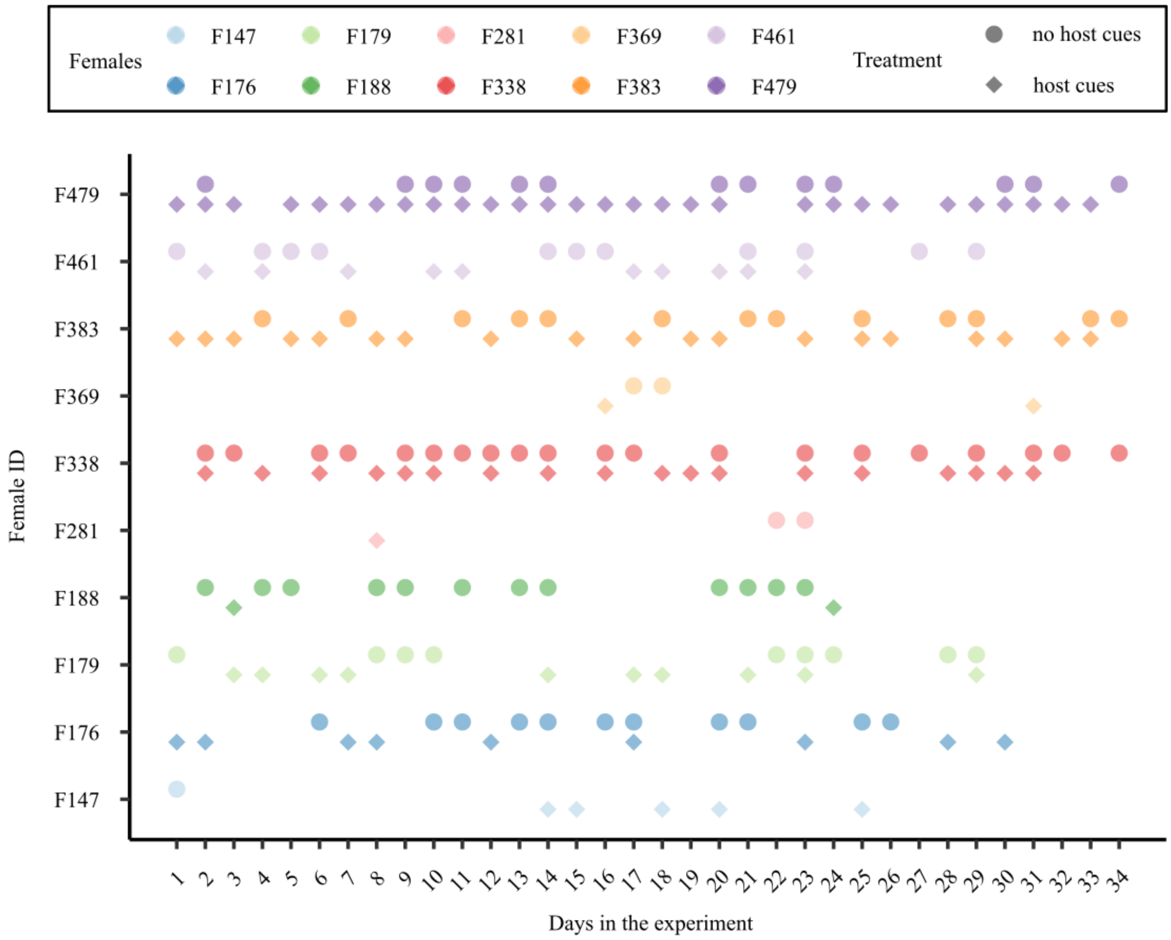

Supplementary Figure S2: Comparison of cuckoo catfish egg release with (dots) and without (diamonds) host cues.

Supplementary Table S1: Quality check for the set of microsatellite loci used for parentage analysis (n = 17 loci). Note that significant deviations from Hardy-Weinberg equilibrium (HWE) are expected due to confined population size, non-random mating groups, and relatedness among individuals. A Holm correction was used to correct for multiple testing of deviations from HWE (HWE P adjusted). He = expected heterozygosity, Ho = observed heterozygosity.

Microsatellite Locus	He	Ho	HWE P	HWE P adjusted	Rate of Missing Alleles per locus
Smu_228	0.851	0.844	0	0	0
Smu_071	0.83	0.922	0.002	0.014	0
Smu_456	0.892	0.964	0	0	0
Smu_012	0.807	0.802	0.015	0.09	0
Smu_375	0.715	0.778	0.061	0.3	0
Smu_085	0.604	0.653	0.028	0.161	0
Smu_136	0.836	0.862	0.007	0.036	0
Smu_280	0.51	0.503	0.008	0.09	0
Smu_198	0.786	0.874	0.002	0.014	0
Smu_006	0.809	0.886	0.244	0.456	0
Smu_408	0.871	0.922	0.005	0.09	0
Smu_481	0.809	0.85	0.055	0.3	0
Smu_308	0.769	0.826	0.021	0.161	0
Smu_318	0.861	0.939	0	0	0.012
Smu_185	0.409	0.449	0.002	0.044	0
Smu_357	0.872	0.958	0.127	0.369	0
Smu_328	0.297	0.311	0.681	0.706	0

Supplementary Table S2: Cuckoo catfish clutches used for parentage analysis and their reconstructed parent structure. MZ: *Metriaclima zebra*, GP: *Jabarichromis (Gnatochromis) pfefferi*.

Tank ID	Clutch ID	Host	Sampling date	Number of catfish eggs	Number of parental males	Number of parental females
EXP3-01	#8	MZ	10.10.2022	1	1	1
EXP3-01	#6	MZ	14.10.2022	15	1	1
EXP3-01	#7	MZ	21.10.2022	20	1	2
EXP3-01	#9	MZ	09.01.2023	3	1	1
EXP3-01	#4	MZ	11.01.2023	13	1	2
EXP3-01	#5	MZ	11.01.2023	14	2	3
EXP3-01	#3	MZ	10.02.2023	3	2	1
EXP3-01	#2	MZ	13.02.2023	3	1	1
EXP3-01	#10	MZ	06.03.2023	5	1	1
EXP3-01	#11	MZ	06.03.2023	2	1	1
EXP3-01	#12	MZ	13.03.2023	10	2	2
EXP3-01	#1	MZ	15.03.2023	4	1	1
EXP3-01	#13	MZ	15.03.2023	3	1	1
EXP3-01	#25	MZ	11.04.2023	12	1	1
EXP3-01	#26	MZ	02.05.2023	5	2	2
EXP3-02	#14	MZ	20.01.2023	2	1	1
EXP3-02	#15	MZ	06.02.2023	1	1	1
EXP3-02	#16	MZ	20.03.2023	2	1	1
EXP3-02	#17	MZ	24.03.2023	7	1	1
EXP3-02	#27	MZ	11.04.2023	2	1	1
EXP3-02	#28	MZ	17.04.2023	4	2	2
EXP3-03	#18	MZ	17.10.2022	1	1	1
EXP3-03	#19	MZ	09.01.2023	1	1	1
EXP3-03	#20	MZ	09.01.2023	5	1	1
EXP3-03	#21	MZ	12.01.2023	3	1	1
EXP3-03	#22	MZ	16.01.2023	17	1	1
EXP3-03	#23	MZ	08.02.2023	3	1	1
EXP3-03	#24	MZ	06.06.2023	3	1	1
EXP3-04	#29	MZ	23.01.2023	2	1	1

EXP3-04	#31	MZ	13.02.2023	9	1	1
EXP3-04	#32	MZ	27.02.2023	2	1	1
EXP3-04	#33	MZ	06.03.2023	1	1	1
EXP3-04	#34	MZ	08.03.2023	3	1	1
EXP3-04	#35	MZ	24.03.2023	15	1	2
EXP3-04	#30	MZ	11.04.2023	5	1	1
EXP3-05	#36	GP	19.09.2022	6	1	1
EXP3-05	#37	GP	30.09.2022	1	1	1
EXP3-05	#38	GP	21.10.2022	2	1	1
EXP3-06	#39	MZ	30.09.2022	22	1	1
EXP3-06	#40	MZ	12.10.2022	10	1	1
EXP3-06	#41	MZ	12.10.2022	2	1	1

Supplementary Table S3: Results of a GLMM with betabinomial error distribution to investigate the effects of total body length, body condition, and the frequency of gamete release during the group phase of the gamete release experiment on the distribution of maternity. Significant values in bold.

	Estimate	Std. Error	z value	Pr(>|z|)
(Intercept)	-4.04788	3.084672	-1.312	0.189
Standard length (mean centered)	0.007292	0.128796	0.057	0.955
Gamete release	-0.00762	0.017143	-0.444	0.657
Fulton's CF	2.787014	2.064253	1.35	0.177

Supplementary Table S4: Results of a GLMM with betabinomial error distribution to investigate the effects of total body length, body condition, and the frequency of gamete release during the group phase of the gamete release experiment on the distribution of paternity. Significant values in bold.

	Estimate	Std. Error	z value	Pr(>|z|)
(Intercept)	14.67071	4.20531	3.489	0.0005
Standard length (mean centered)	-0.63474	0.15837	-4.008	< 0.0001
Gamete release	-0.0364	0.01785	-2.04	0.0414
Fulton's CF	-12.0534	4.36757	-2.76	0.0058
